# Transcultural Adaptation and Psychometric Validation of the Spanish Version of the Pain Attitudes and Beliefs Scale for Physiotherapists

**DOI:** 10.3390/jcm12186045

**Published:** 2023-09-19

**Authors:** Ángeles Díaz-Fernández, Ana Raquel Ortega-Martínez, Irene Cortés-Pérez, Alfonso Javier Ibáñez-Vera, Esteban Obrero-Gaitán, Rafael Lomas-Vega

**Affiliations:** 1 Department of Health Sciences, University of Jaen, Campus las Lagunillas, 23071 Jaen, Spainrlomas@ujaen.es (R.L.-V.); 2 Department of Psychology, University of Jaen, Campus las Lagunillas, 23071 Jaen, Spain

**Keywords:** low back pain, attitude, physical therapists, psychometrics, beliefs scale

## Abstract

Low back pain (LBP) is one of the main musculoskeletal pain conditions, and it affects 23–28% of the global population. Strong evidence supports the absence of a direct relationship between the intensity of pain and tissue damage, with psychosocial factors also playing a crucial role. In this context, the Pain Attitudes and Beliefs Scale for Physiotherapists (PABS-PT) is a useful tool for evaluating physiotherapists’ treatment orientations and beliefs regarding the management of low back pain (LBP). It helps identify practitioners who may benefit from additional education in modern pain neuroscience. However, there is not a Spanish validation of this scale for physiotherapists. Thus, the aims of this study were to translate and culturally adapt the Pain Attitudes and Beliefs Scale for Physiotherapists (PABS-PT) into Spanish and to evaluate its psychometric properties. This validation study used three convenience samples of physiotherapists (PTs) (*n* = 22 for the pilot study, *n* = 529 for the validity study and *n* = 53 for assessing the instrument’s responsiveness). The process of translating and adapting the PABS-PT into Spanish followed international guidelines and produced a satisfactory pre-final version of the questionnaire. Factor analysis confirmed the two-factor structure of the original version, with the biomedical (BM) factor explaining 39.4% of the variance and the biopsychosocial (BPS) factor explaining 13.8% of the variance. Cronbach’s alpha values were excellent for the BM factor (0.86) and good for the BPS factor (0.77), indicating good internal consistency. Test–retest reliability was excellent for both factors, with intraclass correlation coefficients (ICCs) of 0.84 for BM and 0.82 for BPS. The standard error of measurement (SEM) was acceptable for both factors (3.9 points for BM and 2.4 points for BPS). Concurrent validity was moderate and in the expected direction and had significant correlations with the Health Care Providers’ Pain and Impairment Relationship Scale (HC-PAIRS) and Revised Neurophysiology Pain Questionnaire (R-NPQ). Sensitivity to change was demonstrated by significant improvements in both factors after an educational intervention, with medium-to-large effect sizes. The PABS-PT also showed good discriminative ability, as it was able to distinguish between physiotherapists with and without pain education. Cut-off values for the BM and BPS factors were determined. In conclusion, the translated and adapted Spanish version of the PABS-PT demonstrated good psychometric properties and can be reliably used to assess the attitudes and beliefs of Spanish-speaking physiotherapists regarding LBP. The questionnaire is recommended for use in clinical and educational research in the Spanish language context.

## 1. Introduction

Non-specific low back pain (LBP) is a widely recognised medical issue that affects individuals globally and has significant economic implications [1,2,3,4]. Studies have shown that approximately 23–28% of the general population experiences LBP [5,6], with a prevalence of 19.9% in Spain specifically [7]. It is expected that most individuals will have at least one episode of LBP in their lifetime, and around one third of these episodes will progress to a chronic condition, leading to disabilities for about 11–12% of people [5,6]. In raw numbers, in 2019, there were approximately 223.5 million reported cases of low back pain (LBP) worldwide, leading to 63.7 million disability-adjusted life years (DALYs) associated with LBP [8]. Interestingly, the level of disability related to LBP is highly variable and cannot be solely explained by the intensity of the pain or the extent of tissue damage [9]. Compelling evidence indicates a weak correlation between pain and structural changes or biomedical findings [3]. Furthermore, symptoms of LBP can persist over prolonged periods even in the absence of severe disease, highlighting the critical role of the psychosocial component in the development of LBP-related disability and chronicity [10]. Despite these facts, physiotherapy management options are still insufficient [11,12], and chronic low back pain (CLBP) costs have not decreased [13]. Treatment often aims to reduce pain-related fear avoidance behaviour and recover functioning, including returning to work. One proposed solution is implementing a multimodal approach incorporating psychological, cognitive, and behavioural strategies into treating LBP [12,14].

In recent years, significant advancements have been made in our understanding of the neurophysiology of pain, particularly chronic pain. However, numerous rehabilitation professionals have not implemented these breakthroughs in their clinical practices, especially regarding rest advice, passive treatment methods, and limiting physical activities [15]. Research has shown a connection between the attitudes and beliefs of healthcare providers (HCPs) and their clinical decisions, treatment preferences, and recommendations for patients with CLBP [16,17,18,19,20,21,22]. The beliefs of HCPs can also influence the beliefs of their patients about pain [17,23,24]. Moreover, the attitudes and beliefs of HCPs may impact patient outcomes [18,25]. By addressing and changing these beliefs and attitudes, we can improve therapeutic outcomes, especially in the field of physiotherapy [18,26]. Unfortunately, limited studies are focusing on this perspective. Standardised measurements with validated psychometric properties suitable for the target population are required to assess these essential aspects.

The Pain Attitudes and Beliefs Scale for Physiotherapists (PABS-PT) [27] is a commonly used self-administered questionnaire for assessing the attitudes and beliefs of physical therapists in the development and maintenance of LBP [28]. It distinguishes between a biomedical and biopsychosocial treatment approach to managing persistent LBP. The PABS-PT is widely accepted in the scientific community thanks to its two-dimensional structure. Multiple studies have assessed the PABS-PT and, except for one study [29], consistently found a two-factor solution in the factor analysis. However, the measurement properties of the PABS-PT have received criticism, even from the original researchers, especially regarding the biopsychosocial factor. Some studies have suggested that the instrument is still in the process of development and refinement. The availability of a questionnaire in multiple languages ensures that research findings can be applied to a broader population. The researchers originally developed a Dutch version of the PABS-PT questionnaire and also provided an English translation in their publications [27,30]. Since then, the questionnaire has been adapted into several more languages, including German [31], Brazilian Portuguese [15], Turkish [32], and Norwegian [33]. However, to our knowledge, no validation of the questionnaire’s psychometric properties has been conducted for the Spanish PTs population. Other studies in different contexts and for diverse HCPs populations have also utilised the PABS-PT questionnaire [34,35].

Based on these facts, the main objective of this study was twofold: first, to translate and culturally adapt the PABS-PT questionnaire from English to Spanish (PABS-PT-SP) and second, to assess its psychometric characteristics in a sample of Spanish physiotherapists (PTs).

## 2. Materials and Methods

### 2.1. Participants and Design

The study was approved by the Institutional Ethics Committee of the Andalusian Health Service (Jaen, Spain) under the code n° 280612, and all procedures were conducted in accordance with the Declaration of Helsinki [36]. The responders’ written consent was obtained before the study onset. The PTs were instructed that the purpose of the questionnaire was to assess how they approach the management of non-specific LBP, which does not include back pain resulting from radicular syndrome, cauda equina syndrome, fractures, infections, inflammation, a tumour or metastasis.

**Figure 1:**
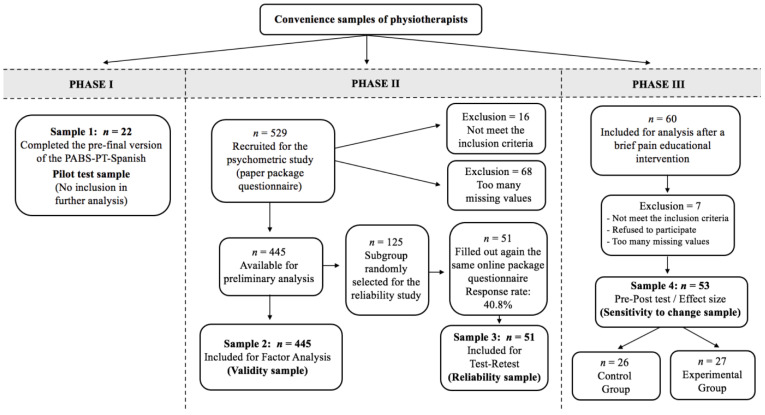
Flow diagram.

The study followed the COSMIN study design checklist for patient-reported outcome measurement instruments [37] and the COSMIN reporting guidelines for studies on the measurement properties of patient-reported outcome measures [38]. The study’s sample sizes were considered appropriate according to established psychometric guidelines, which recommended that at least 50 participants would be necessary for the reproducibility, validity and ceiling and floor effects analysis and that at least 100 participants would be needed for the internal consistency analysis [37]. The sole inclusion criteria for participation in the study was that the PTs should have treated patients with LBP in the last six months (this information was collected by asking a specific question in the survey). The study was conducted in three phases using various convenience samples of PTs. In the first phase, the English PABS-PT [30] was translated and adapted into a Spanish version called PABS-PT-SP. The second phase involved evaluating the factor structure and psychometric properties of the PABS-PT-SP. In the third phase, the responsiveness of the new instrument was analysed. Figure 1 shows the flowchart of the study.

#### 2.1.1. Phase I: Translation and Cross-Cultural Adaptation

**Figure 2:**
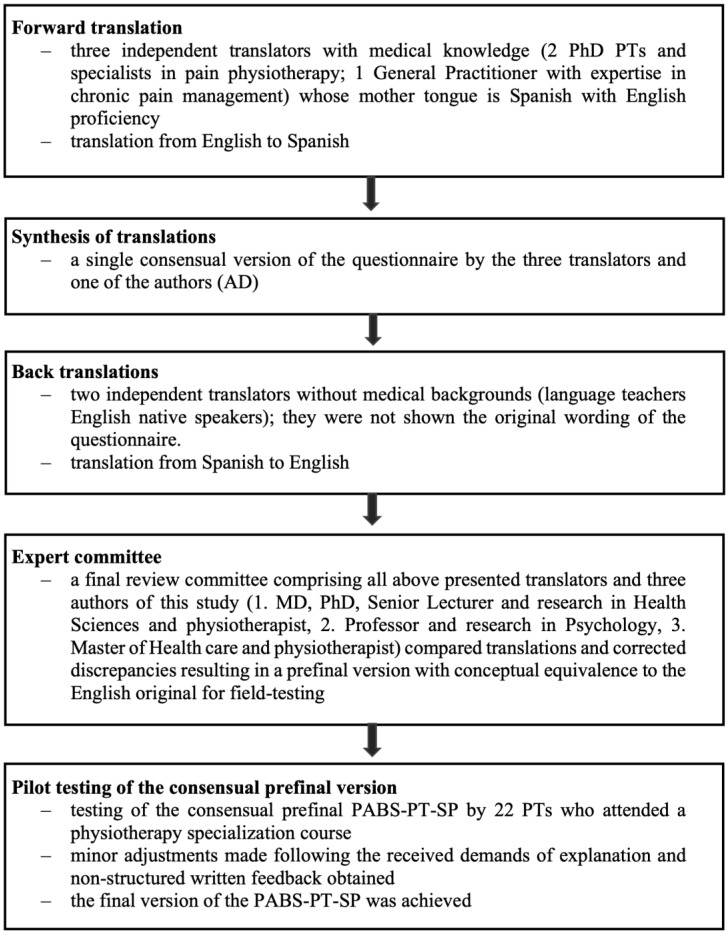
Transcultural adaptation process.

One of the developers, Ostelo R., gave authorization to translate the PABS-PT questionnaire from English to Spanish. All 36 items included in the Dutch study by Houben et al. [27,30] were translated into Spanish following established international guidelines [37,39] for self-reported measurement instruments. The translation process consisted of five steps, as outlined in Figure 2.

#### 2.1.2. Phase II: Investigation of Psychometric Properties

Within a cross-sectional study design, a second convenience sample of 529 participants was recruited through three channels. Data were collected from (1) private physiotherapy companies and rehabilitation institutions in the south of Spain (covering ~100 PTs); (2) PTs attending different specialization courses at four universities of health sciences in Spain (covering ~500 PTs); and (3) PTs from seven public hospitals in the south of Spain (covering ~120 PTs). They were asked to fill out on paper a packed questionnaire that was composed of the PABS-PT-SP, the Health Care Providers’ Pain and Impairment Relationship Scale (HC-PAIRS) [16], the Revised Neurophysiology Pain Questionnaire (R-NPQ) [40] and sociodemographic questions. The researchers only had access to the PTs’ email addresses and no other personal data. The recruitment took place from October 2019 to May 2022.

A randomly selected group of participants (*n* = 125) received the packed questionnaire via email. They were asked to complete the survey a second time within a time frame of 48 hours to 1 week [41]. This interval was chosen to prevent recall bias from the first response and to ensure that there was insufficient time for their beliefs about LBP to change. A reminder email was sent after five days, and online participation closed after seven days.

#### 2.1.3. Phase III: Investigation of Sensitivity to Change

The 60 PTs recruited for this convenience sample attended a seminar unrelated to chronic musculoskeletal pain. Using SPSS, the sample was randomly divided into two groups (control group, *n* = 26, and experimental group, *n* = 27). Baseline data were collected, and seven days later, the experimental group received a fifteen-hour neuroscience university-accredited course on pain. This training included information on the neurobiology and neurophysiology of pain and pain processing by the nervous system, and it was designed to enhance knowledge and management of psychosocial factors in their practice with patients suffering from persistent musculoskeletal pain [42]. Both groups completed the questionnaire on paper again after the training, with the control group receiving the same training ten days later.

### 2.2. Outcome Measures

#### 2.2.1. Sociodemographic Data

Sociodemographic and professional data were collected from the participants. These included gender, age, number of years working, employment status, experience in treating LBP patients in the past six months, and whether they had received previous specific pain education courses.

#### 2.2.2. Pain Attitudes and Beliefs Scale for Physiotherapists

The original 36-item PABS-PT [27] was further examined by Houben et al. [30], which resulted in a shorter 19-item version. PTs were asked to rate the statements on a 6-point Likert scale ranging from ‘*totally disagree*’ to ‘*totally agree*’. The total score of the biomedical subscale ranges from 10 to 60 points (10 items), and the total score of the biopsychosocial subscale ranges from 9 to 54 points (9 items). A high score on the first factor represents conviction in the relationship between pain and structural damage. On the contrary, a high score on the second factor signifies a lack of confidence in this relationship. It reflects the belief that despite experiencing pain, it is still possible to overcome functional limitations. The psychometric properties of the questionnaire have been thoroughly reviewed and were found to be satisfactory [43]. However, the instrument developers noted that the PABS-PT was ‘*still at a development stage*’ and, especially the biopsychosocial factor, was open to improvement (e.g., by adding items) since the internal consistency of this factor was only just acceptable. They did not establish a clear threshold for determining high or low scores on any factor. Furthermore, the two-factor structure has also been questioned, and more investigation for content validity and internal consistency was suggested [29].

#### 2.2.3. Health Care Providers’ Pain and Impairment Relationship Scale

Rainville et al. [16] adapted the HC-PAIRS from the Pain and Impairment Relationship Scale (PAIRS) initially developed for people with LBP. The HC-PAIRS assesses the attitudes and beliefs of healthcare providers about LBP and serves as a predictor for clinical recommendations regarding activity and work. It was revised by Houben et al. [44] from 15 to 13 statements. Each item is counted on a seven-point Likert scale (‘*totally disagree* = 1’ to ‘*totally agree* = 7’) with three items being reverse scored. Thus, scores can range from 13 to 91, with a higher grade on the HC-PAIRS indicating stronger belief that LBP requires avoiding activities and validates disability. The HC-PAIRS has a one-dimensional structure and measures only the biomedical treatment orientation of HCPs. In our study, we used the Spanish version of the HC-PAIRS [45], which has previously been translated and cross-culturally adapted and presented adequate psychometric properties (Cronbach’s alpha = 0.82; ICC = 0.50).

#### 2.2.4. Revised Neurophysiology Pain Questionnaire (R-NPQ)

The Neurophysiology of Pain Questionnaire (NPQ) was initially developed by Moseley et al. [46] to assess postgraduate medical students’ understanding of the biology of pain. The language of the original NPQ has been adapted for patients and is used both in clinical practice and in research [47,48,49,50]. The original 19-item questionnaire was revised by conducting a Rasch analysis: acceptable internal consistency (person separation index = 0.84) and test–retest reliability were reported (ICC = 0.97) in patients with chronic pain, but Seven items were found to have suboptimal functionality, thus adversely affecting the psychometric properties [40]. This revised version (R-NPQ) is a valid and reliable tool for assessing the neurophysiology of pain. Each item has a true, false or undecided response; correct responses were given 1 point, and incorrect (or undecided) responses were given 0 points. Therefore, the result is determined by adding up the number of correct answers, and the overall score can range from 0 to 12, with higher scores indicating greater knowledge about pain.

Our study utilised the Spanish version of the R-NPQ [51], which had already been translated and cross-culturally adapted for use with patients and PTs. This version demonstrated satisfactory psychometric properties (Cronbach’s alpha = 0.90; ICC = 0.82).

### 2.3. Statistical Analyses

All data management and analysis were performed using IBM SPSS Statistics for Macintosh, Version 27.0 (IBM Corp., Armonk, NY, USA). The level of statistical significance was set at *p* < 0.05. Descriptive data were shown as percentages or means with standard deviations (SD). All data were examined for normality through the Kolmogorov–Smirnov test with the Lilliefors correction.

*Structural validity* was assessed using exploratory factor analysis (EFA) in the principal sample (*n* = 445). The analysis followed a procedure similar to the one suggested by Houben et al. [30,44] to allow for comparison. However, more restrictive criteria were used because the instrument was still in the developmental stage, and the factors were not yet clearly defined.

Before examining the factor structure of the PABS-PT-SP, the psychometric properties of each item were analysed; we excluded items for heterogeneity (if skewness or kurtosis exceeded ±1). During the EFA, items with a communality score < 0.40 were deleted. We also excluded items with factor loadings below 0.5 in order to clearly define the two expected factors and avoid any potential confusion with other hiding factors.

Additionally, if loading on one factor exceeded 0.5 but differences between loadings were less than 0.1, items were also excluded. The Kaiser–Meyer–Olkin (KMO) and Bartlett sphericity tests were performed in order to decide whether the sample was suitable for the EFA. A principal factor analysis with oblique rotation (Oblimin with Kaiser normalisation) was prioritised since it was assumed that there was correlation among the factors.

The requirements for extraction were Kaiser’s criterion (eigenvalues > 1), the underlying theory and Cattell’s scree plot.

*Internal consistency* of the questionnaire was assessed using the Cronbach’s alpha of items included in each factor and item–total correlation. An accepted guideline for its value is between 0.60 and 0.90 [52]. Item–total correlations should be above 0.40, and lower values indicate that the corresponding item does not correlate well with the scale overall and may be discarded [53]. Pearson’s correlation coefficient between the factors was also calculated.

Test-retest reliability was assessed by analyzing floor and ceiling effects and calculating the intraclass correlation coefficient (ICC) using a two-way mixed-effects model (ICC3,2) with absolute agreement [54]. The values were interpreted: > 0.75 was good, 0.75–0.50 was moderate, and < 0.50 was poor [55]. In order to analyse the level of accuracy, the standard error of measurement (SEM) was calculated (*SEM = SD ×* $\sqrt{1-ICC}$), where SD = standard deviation of the measure at baseline, and ICC corresponds to the ICC obtained from the test–retest reliability calculation of each factor [56]. Floor and ceiling effects were considered to be present if 15% or more of the participants achieved the minimum or maximum scores, respectively, in each factor. The ceiling and floor effects compromise the reliability of the questionnaire, as it becomes difficult to differentiate participants who obtained very low or high scores [57].

Bland–Altman plots were used to assess the questionnaire’s reproducibility and agreement by graphically representing each pair of measurements’ difference and the average score for both factors [58]; they were used to evaluate the systematic differences by generating the 95% limits of agreement (LOAs).

*Interpretability* was assessed using the minimal detectable change (MDC), thereby determining the minimum statistically significant difference in measurement results [59]. It was calculated using the formula MDC_95_ = 1.96 × √2 × SEM. If the observed change exceeds the MDC value, then it will likely be a true change rather than a measurement error.

*Concurrent validity* was measured in the principal sample using Pearson correlation coefficients (*r*) between the total score of each factor of the PABS-PT-SP and the total score of the HC-PAIRS and R-NPQ in the absence of a proper gold standard. An *r* value <0.30 indicates a weak correlation, *r* values ≥ 0.30 and < 0.60 indicate a moderate correlation, and *r* values ≥ 0.60 indicate a strong correlation [60,61,62]. We expected a positive association of the BM factor of the PABS-PT-SP with the HC-PAIRS (moderate correlation) and a negative association with the R-NPQ (weak correlation), as they measure similar concepts but are not identical. Conversely, we anticipated a similar correlation strength of the BPS factor of the PABS-PT-SP with those instruments but in the opposite direction.

*Discriminative validity* was assessed by known-groups validity in both factors of the instrument [63]. Before analysis, two groups were formed by crossing the scores of both PABS-PT-SP factors with the baseline variables for hypothesis testing. This was done under the assumption that there would be a significant difference in PABS-PT-SP scores for both factors in relation to receiving specific pain education. This assumption was based on the premise that implementing pain neuroscience training enhances biopsychosocial thinking [64,65]. Comparisons between groups were conducted using an independent-sample *t*-test. In this context, a value of 0.20 would indicate a small effect size, a value of 0.50 would suggest a medium effect size, and a value of 0.80 would represent a large effect size [66].

*Sensitivity to change:* To evaluate the instrument’s ability to detect participant improvements, a paired *t*-test was performed for each factor, comparing the control and experimental groups. Previous research suggested that a pain education intervention would significantly impact both factors, particularly the BPS factor measured by the PABS-PT-SP [67,68]. A large effect was anticipated. For the assessment of responsiveness, we calculated the standardised response mean (SRM) as the mean change score divided by the SD of the change score [69]. Values of 0.20–0.49 were considered small, 0.50–0.79 medium and 0.8–1.0 large effects [70]. However, as there is no consensus on the precise measurement of change over time, we conducted two additional effect size calculations in order to provide a more comprehensive evaluation of the intervention’s impact. These calculations involved assessing the Pearson correlation coefficient (*r*) and the coefficient of determination (*R*^2^) between the two factors in the pre- and post-intervention assessments. By considering both underlying factors simultaneously and quantifying how their correlation changes over time, these measures could provide a more robust evaluation of the effect size.

*Predictive ability* was determined using receiver operating characteristic (ROC) curves and area under the curve (AUC) analyses. The curves were constructed using the total scores from the PABS-PT-SP and self-reported pain education as the criteria since no true gold standard was available to classify the participants properly. It was assumed that individuals who had attended prior pain courses would have a higher degree of pain knowledge. The optimal cut-off values for detecting a weakening in pain knowledge were determined by examining the intersections of the sensitivity and specificity plots closest to the upper left corner of the graph. AUC was considered statistically significant when the 95% confidence interval (CI) did not include the 0.5 value [71].

## 3. Results

### 3.1. Demographic Characteristics and Responses of the Instruments

The principal sample for the factor analysis comprised 529 participants. Sixteen participants did not meet the criterion of having treated LBP patients within the last six months, twelve returned a blank PABS-PT-SP questionnaire without providing a reason, and fifty-six participants had three or more missing values in the questionnaires and were excluded. However, 26 participants had only one or two missing values that did not follow a specific pattern, so they were included in the analysis. The missing values in these cases were filled with the middle score value of the scale (3, 4 or 1, respectively). Therefore, the final sample used for validity analysis included 445 participants. Fifty-one PTs from the random subsample (response rate 40.8%) completed the retest. Of the initial 60 PTs in the sensitivity to change sample, seven were excluded for various reasons: five had not treated LBP patients in the last six months, two refused to participate, and one questionnaire had almost no data; the remaining 53 PTs completed the questionnaires after the pain educational intervention.

The sample characteristics and baseline test scores of the questionnaires are presented in Table 1.

### 3.2. Translation and Cultural Adaptation

The pre-final version of the test was considered satisfactory. Twenty-two participants completed the PABS-PT-SP questionnaire. Their feedback indicated that the questionnaire was clear and easy to understand. However, some respondents mentioned that the total survey was lengthy. Based on their feedback, minor changes were made to two items (22 and 29). No data were missing, and the completion time ranged from 2 to 7 min. This version was used in the validation study without further modification.

### 3.3. Construct Validity

**Figure 3:**
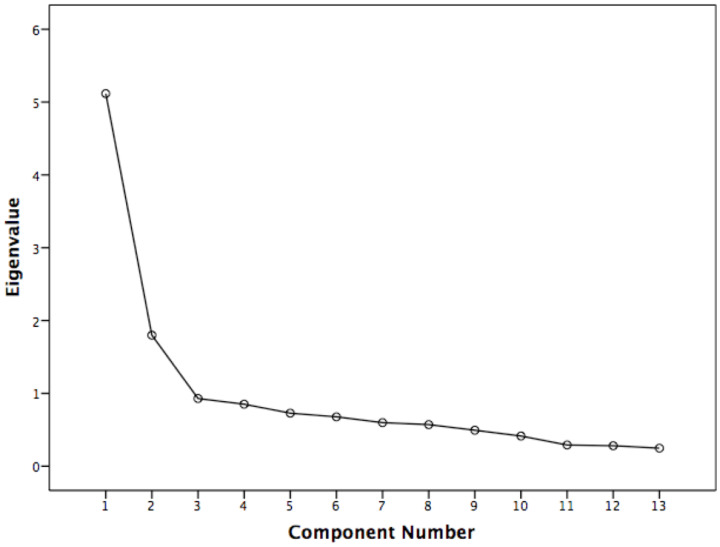
Scree plot.

Before factor analysis, three items were excluded because of non-heterogeneity (skewness or kurtosis exceeded ±1). A KMO measurement of 0.784 and Bartlett’s test of sphericity (χ^2^ = 6926.05; *p* ≤ 0.001) justified continuation of the analysis. A principal factor analysis with Oblimin rotation and Kaiser normalisation was performed. The eigenvalue > 1 criterion initially yielded 11 factors. Eighteen items were discarded because of factor loadings below 0.5, and two items were removed because of a rise in alpha if deleted. The eigenvalues > 1 criterion now showed two factors; the scree test supported this assumption (Figure 3). The explained variance was 53.2% (39.4% of the BM factor and 13.8% of the BPS factor). Table 2 shows the descriptive data for all items, reasons for exclusion, factor loading and factor composition for the included items. There was a moderate negative correlation between the two subscales of the PABS-PT-SP (*r* = −0.45; *p* < 0.01).

### 3.4. Internal Consistency

The Cronbach’s alpha was excellent for the BM factor—0.86 (8 items)—and good for the BPS factor—0.77 (5 items). The total–item correlations were above 0.40 and ranged between *r* = 0.56 and *r* = 0.7 for the BM factor and *r* = 0.47 and *r* = 0.64 for the BPS factor.

### 3.5. Test–Retest Reliability and Interpretability

**Figure 4:**
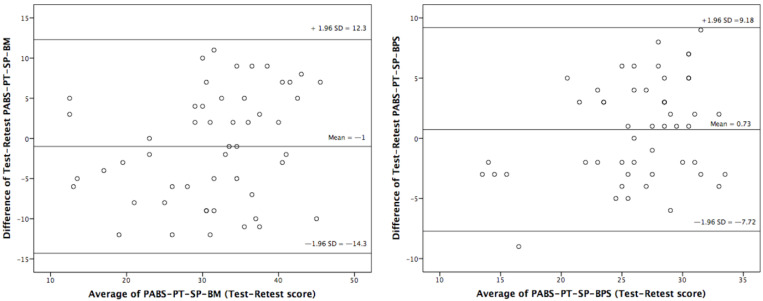
Bland-Altman 95% limits of agreement (LOAs) of the PABS-PT-SP both in the Biomedical and Biopsychosocial factors in physiotherapists, (n = 51)..

Test–retest reliability was excellent for both factors, showing an ICC = 0.84 (95% confidence interval (CI): 0.72–0.91) for the BM factor and an ICC = 0.82 (95% CI: 0.67–0.89) for the BPS factor. The SEM was acceptable for both factors: 3.3 points for the BM factor and 2 points for the BPS factor. We observed no floor or ceiling effects. Bland–Altman plots in Figure 4 indicate good reproducibility of the new instrument version, as the differences for the PABS-PT-SP were centred around zero on both subscales (no bias indicated). For interpretability, the MDC_95_ was 8.8 points and 5.5 points for the BM and BPS factors, respectively.

### 3.6. Concurrent Validity

Concurrent validity showed moderate agreement with the HC-PAIRS and was consistent with the expected direction for each subscale (*r* = 0.48; *p* < 0.01 for the BM factor, and *r* = −0.57; *p* < 0.01 for the BPS factor). It was also moderate with the R-NPQ (*r* = −0.38; *p* < 0.01 for the BM factor, and *r* = 0.41; *p* < 0.01 for the BPS subscale). The hypotheses were verified, although with fewer robust associations than anticipated.

### 3.7. Discriminative Ability (Known Groups Validity)

The comparison of the group scores (PTs with specific pain education versus PTs without pain education) was significant for the BM factor (*t* = −2.266; *gl* = 443; *p* < 0.05) with a small effect (*d* = 0.46 [95% CI: 0.21–0.58]), but not for the BPS factor (*t* = 1.284; *gl* = 443; *p* = 0.182). The previous hypothesis, therefore, was not confirmed completely.

### 3.8. Sensitivity to Change and Responsiveness

The scores of the 27 participants selected for the pain educational training did not differ significantly from the controls on pre-intervention measures (*p >* 0.05). The sensitivity to change was significant in both factors: the BM factor (*t* = 3.231; *gl* = 26; *p* < 0.05) with a medium effect (SRM of 0.53 [95% CI: 0.22–0.73]), and the BPS factor (*t* = −3.567; *gl* = 26; *p* < 0.001) with an almost large effect (SRM of 0.76 [95% CI: 0.39–0.98]). Pearson’s correlation coefficients between the two factors were consistently similar across all conditions, showing a moderate negative correlation. However, in the experimental group, after the intervention, the correlation coefficient changed from moderate to a strong negative correlation (*r* = −0.68; *p*-value < 0.001), supporting the expected direction and magnitude of the intervention. Additionally, the coefficient of determination *(R^2^)* doubled after the educational intervention (see Table 3).

### 3.9. Predictive Ability

**Figure 5:**
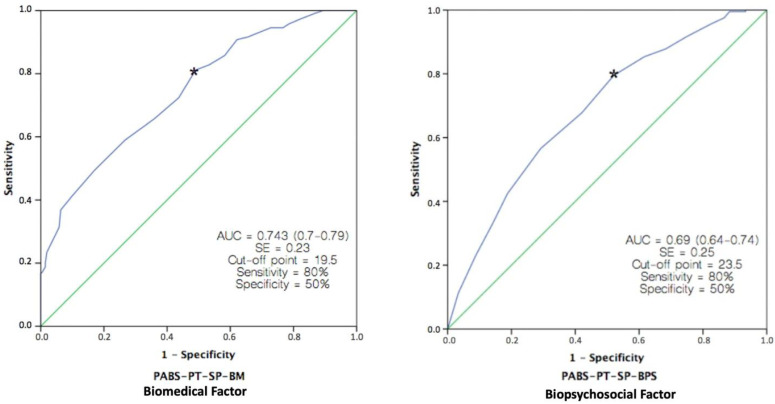
Receiver operating curves (ROC) for the PABS-PT-SP Biomedical and Biopsychosocial factors against an external criterion (Specific pain education). n = 445. The green line represents the non-discrimination line. Asterisks mark the cutoff point that maximizes the combined sensitivity and specificity for both factors: 0.74 for BM; 0.69 for BPS.

ROC curves were used to determine the accuracy of the BM and BPS factors in predicting knowledge about pain. The area under the curve (AUC) values were 0.74 for the BM factor and 0.69 for the BPS factor, indicating fair discriminative ability for the BM factor and poor discriminative ability for the BPS factor. The optimal cut-off values, which defined the clinically important difference for this ability, were 19.5 points for the BM factor and 23.5 points for the BPS factor. Figure 5 visually represents the AUC values and the sensitivity and specificity data for these cut-off values.

## 4. Discussion

This study aimed to translate and adapt the PABS-PT scale into Spanish and to evaluate its psychometric properties in a Spanish sample of physical therapists (PTs). The translation and adaptation process was straightforward, resulting in a Spanish version of the questionnaire that was deemed to be equivalent to the original PABS-PT. The analysis supported the two-factor structure of the instrument, as previously reported, and shortened the total scale to 13 items. Overall, this study successfully translated and adapted the PABS-PT scale for use in a Spanish-speaking population of PTs and provided a validated instrument for assessing professional attitudes and behaviours both in clinical and educational settings.

Recently, a study validated the PABST-PT questionnaire in Spanish physiotherapy students [72]. Although this instrument has also been used in this population for educational purposes, the ideal target population for accurately measuring attitudes and beliefs about pain and patients with prolonged pain should be professionals with theoretical knowledge and postgraduate clinical experience with those patients. For this reason, the selected sample in our study consisted only of PTs, and the inclusion criteria were explicitly based on having treated patients with LBP in the last six months.

In this study, an exploratory factor analysis (EFA) was used instead of a confirmatory factor analysis (CFA) because the scale is still under development, and there is no uniformity in the choice of items in different cross-cultural validations. Additionally, internal consistency is still lacking, especially in the BPS factor. EFA allows for an initial factor solution with few restrictions, which can then be transformed through rotation. On the other hand, CFA is more restrictive and evaluates the fit of a single solution using different fit indices. However, the CFA model makes assumptions that are not realistic in many cases, such as the restriction that item loadings in supposedly unrelated factors be zero, especially when the factors are correlated, as in the PABS-PT [73]. This study aimed to identify the items that best characterize the two factors previously defined by theory: the BM and BPS factors. The goal was to obtain major factors or markers and their most representative items [73,74]. To achieve this, stricter criteria were used in the EFA to eliminate items, such as item heterogeneity values (±1), communalities (>0.4) and loadings on factors (>0.5). Various criteria were employed for factor retention, including eigenvalues greater than one, the underlying theory and the scree plot (visual analysis further validated the two-factor solution by excluding problematic items). The internal consistency of the BM factor was higher than the BPS factor, which is consistent with previous studies. The Cronbach’s alpha for the BM factor (α = 0.86) was satisfactory and in line with the range of 0.72–0.83 reported in other investigations. The Cronbach’s alpha for the BPS factor reached recommended levels (>0.7), specifically 0.77. Along with Mutsaers et al. [41] and Gacto–Sánchez et al. [72]*,* these are the only studies in which the internal consistency of the BPS factor obtains such a high value. However, we still believe that this scale does not sufficiently reflect this dimension due to the low cumulative variance explained (13.8%) and the heterogeneity of selected items observed in other studies. The construct of the “biopsychosocial” complex should remain open to improvement, potentially by including additional items and/or analyzing other underlying factors. Table 4 compares studies examining the factor structure and internal consistency of the PABS-PT. Supplementary File S1 comprehensively compares the specific items used in our instrument and those identified by other researchers. Globally, the most shared items for Factor 1 (*Biomedical*) were items 10, 20, 25, 30 and 31, while items 6, 11 and 12 were the most commonly shared for Factor 2 (*Biopsychosocial*), indicating some agreement across studies. However, there were also differences in the items included, suggesting that there is still room for improvement in the scales. The final version of the PABS-PT-SP is described in Supplementary File S2.

Table 4 provides a concise summary of the factorial structure and internal consistency of various studies on the PABS-PT. For a more in-depth understanding, a comprehensive overview of the psychometric properties and results from these studies on the PABS-PT is shown in Supplementary File S3.

The reliability of our measure was found to be excellent for both factors, with intra-class correlation coefficients (ICC) of 0.84 for the BM factor and 0.82 for the BPS factor. These values were consistent with, and only slightly higher than, those reported by previous studies. It is worth noting that only five of these previous studies reported reliability [15,31,32,41,72]. We also found no evidence of floor or ceiling effects, meaning that our questionnaire could distinguish between PTs with the lowest or highest possible score [75]. We used Bland–Altman plots to further assess the reproducibility between measures and calculated the limits of agreement (LOAs). Our results showed good agreement, as the zero point was within the 95% confidence interval for both factors, and 96% of the scores in both factors fell within the 95% LOAs. These findings were consistent with a previous study [41] that used Bland–Altman plots and reported similar results, indicating good agreement and no trend in the points. The SEM (standard error of measurement) was used to evaluate the instrument’s accuracy. The values obtained were deemed acceptable for both factors, comparable to those found in previous studies conducted on the Brazilian Portuguese version [15] and for the *‘neck pain’* version [41]. The values were slightly lower than those reported in the survey of Spanish physiotherapy students [72], which could possibly be attributed to using different sample types. We are unaware if any other studies have measured the SEM for both factors of the instrument.

When interpreting research findings within a defined period, researchers must consider the significance of any changes observed in a measured variable or outcome. The minimal detectable change (MDC) is a calculated value used to assess the significance of such changes. However, only two studies have investigated this statistical estimate, the least-evaluated psychometric characteristic in previous studies. Our findings aligned closely with an investigation by Mutsaers et al. [41] on both factors (BM factor 8.8 vs. 8.3 points; BPS factor 5.4 vs. 4.4 points), although some differences were observed in the students’ physiotherapy study [72] regarding the BM factor (MDC = 6 points). It should be noted that both Mutsaers et al. [41] and our study only included professional PTs. In contrast, the other study consisted of students from a single university, resulting in a more homogeneous sample that could have influenced the estimation of the MDC, potentially leading to an overestimation due to the lower SEM observed.

The concurrent validity of the factors assessed concerning the HC-PAIRS was confirmed, with a correlation coefficient of 0.48 for factor BM and −0.57 for factor BPS. These correlations were higher than those reported by Magalhaes et al. [15] (*r* = 0.28 for factor BM; *r* = 0.19 for factor BPS), where, surprisingly, both factors showed a positive relationship with the instrument. The correlations in our study were similar to those obtained by Houben et al. [30] (*r* = 0.51 for factor BM; *r* = −0.47 for factor BPS), both in terms of magnitude and expected direction. In this study [30], the scores of the PABS-PT were also compared with the *Tampa Scale of Kinesiophobia* (TSK), as in the studies of Laekeman et al. [31] and Dalkilinc et al. [32]. Our study is the first to use the R-NPQ as a comparative measure for concurrent validity. The results confirmed our hypothesis about the direction of the relationship, with a negative correlation of −0.38 for factor BM and a positive correlation of 0.41 for factor BPS. However, the magnitude of these correlations may be smaller than expected because the R-NPQ assesses knowledge about the neurophysiology of pain, which is a different construct from what the factors of the PABS-PT consider.

Regarding discriminant validity (known groups validity), an independent-samples *t*-test revealed statistically significant differences when comparing scores on the BM factor between those who had received specific courses on pain and those who had not, with a small effect size. However, no significant differences were found in the BPS factor. Although the initial hypothesis was logical and plausible, it could not be fully confirmed. This result surprised the research team, as updated knowledge of pain neuroscience is important to attitudes and beliefs about pain in PTs [21,74,75,76]. Our findings contrast with previous studies [72,77], possibly because of differences in the sample (e.g., final-year physiotherapy students who would have received homogeneous, up-to-date, and recent training in pain neuroscience [72]. However, it is essential to note that this study only assessed the discriminative validity of the BPS factor, not the BM factor). Yet, the main reason for the observed results may be the methodology used to measure pain training. The assessment relied solely on participant self-reporting of attendance without considering important factors such as the duration of the training, its specific content, the methodology utilised, or the time elapsed since its completion. Moreover, our study revealed that the instrument identified changes in participants’ pain knowledge following a university-accredited training course. These changes exhibited a moderate effect size for the BM factor and an almost large effect size for the BPS factor, respectively, suggesting that the questionnaire has a discriminative capacity regarding pain knowledge. Hence, the question used to discriminate between PTs with prior knowledge of modern pain neuroscience and those without was not adequately formulated to accurately reflect its presence in our sample. However, future research should explore this property further, including a more objective and comprehensive assessment of pain training.

This PABS-PT validation study is the only one to investigate the sensitivity to change through a longitudinal design that incorporates a pain education intervention. The results confirm that the instrument is able to detect changes in the attitudes and beliefs of PTs, and a significant difference was observed in both the BPS and BM factors. The effect size was medium in the BM factor (SRM = 0.53) and almost large in the BPS factor (SRM = 0.76), indicating that the educational intervention on pain was effective. Previous studies have also used the PABS-PT to evaluate changes in PTs’ attitudes towards pain following educational interventions and found the instrument valid for this purpose [78,79]. Additionally, this study proposes a measure to quantify the effect size more objectively, encompassing both factors together as they are correlated (hypothetically, a perfect score for each factor would result in a maximum increase in this negative correlation). This effect size could be measured by the change in the correlation coefficient (*r*) between the two factors of the PABS-PT instrument before and after the intervention. Additionally, *R^2^* would quantify the change effect’s extent but not indicate its direction.

Our study is the first to assess the predictive ability of the PABS-PT questionnaire. The results suggest that the BM subscale of the questionnaire can moderately identify PTs with insufficient pain knowledge, while the BPS subscale performs poorly in this regard. These findings may be due to an inadequate self-reported question as a criterion for classifying participants, as mentioned above, which is not a reliable standard for evaluating pain knowledge. Consequently, these findings are consistent with those observed for discriminative ability, indicating slightly superior performance for the BM factor when employing the same criterion. Further research using objective and appropriate standards for assessing pain knowledge is necessary to determine whether the questionnaire possesses this property. Therefore, caution is recommended when interpreting the results obtained with the values of the AUC and the cut-off points.

One limitation of our study is that the physiotherapist samples were convenience samples. However, the sample size was large and diverse, and all samples met the requirements for analysis. Therefore, it is important to acknowledge that our findings may not represent the general population, and there may be biases or limitations in the results obtained.

A second limitation of the study in terms of reliability was that data collection was conducted online instead of on paper, as in the rest of the analyses. This method was chosen due to the difficulty of collecting data on paper from randomly selected participants and with the aim of ensuring a high response rate. However, we believe that this did not cause any differences or bias in the results.

A third limitation in our study regarding pain training was the measurement method used, which only considered attendance and did not consider other factors such as the duration, specific content, approach, or time elapsed since completion.

One strength of this study is that it is the first to adapt culturally and analyse the psychometric properties of the PABS-PT questionnaire in Spanish physiotherapists. Furthermore, a thorough analysis of the psychometric properties has been conducted to support the scale’s validity for both clinical and educational use, including two properties that no other study had previously examined. Additionally, our study provides a more appropriate measure of the effect size following a pain educational intervention, one possibly clearer and easier to understand, for a two-dimensional questionnaire in which the factors may be related.

Confirmatory factor analysis could be used in the future to assess the fit of the proposed PABS-PT model to the data. Rasch analysis could be utilised to examine item properties, including difficulty level and discrimination power, which provide insight into the characteristics and contribution of each item to the assessment as a whole. Future regression studies should examine various potential predictors of PTs’ attitudes and beliefs towards treating persistent LBP, taking into account social desirability biases as well as the implicit and explicit attitudes of these HCPs. Furthermore, it would be intriguing to conduct studies that assess not only the attitudes and beliefs of HCPs toward chronic pain but also their competence in managing CLBP and their self-confidence in its management.

## 5. Conclusions

The instrument PABS-PT-SP effectively differentiates between biomedical and biopsychosocial perspectives on LBP among PTs, as indicated by its satisfactory psychometric properties. Additionally, it can be utilised to evaluate the impact of an educational intervention on pain within an academic environment.

The use of PABS-PT-SP is indicated in Spanish-speaking countries.

Additional research is required to enhance and achieve greater consistency in the construct validity, discriminative ability, and predictive validity in light of the findings from this study.

## Figures and Tables

**Table 1:** Sociodemographic characteristics of the participants included in the factor analysis and total scores (*n* = 445).

Variables	Values
Female, n (%)	267 (60)
Age (years), mean (SD, range)	30.1 (6.7, 21–56)
Years of experience, mean (SD, range)	7.2 (6.5, 1–33)
Clinical practice situation, n (%)	
Public	85 (19.1)
Private	234 (52.6)
Freelance	105 (23.6)
Unemployed	21 (4.7)
Clinical experience on LBP in the last six months, n (%)	
Frequently	265 (59.5)
Occasionally	180 (42.5)
Specific pain education, n (%)	189 (42.4)
HC-PAIRS, mean (SD)	57.5 (12.1)
PABS-PT-SP-BM, mean (SD)	21.6 (7.1)
PABS-PT-SP-BPS, mean (SD)	24.1 (4.4)
R-NPQ, mean (SD)	6.8 (2.6)

n: absolute number; SD: standard deviation; LBP: low back pain; HC-PAIRS: Health Care Providers’ Pain and Impairment Relationship Scale; PABS-PT-SP-BM: Pain Attitudes and Beliefs Scale for Physiotherapists Spanish Biomedical Factor; PABS-PT-SP-BPS: Pain Attitudes and Beliefs Scale for Physiotherapists Spanish Biopsychosocial Factor; R-NPQ: Revised Neurophysiology Pain Questionnaire.

**Table 2:** Descriptive data for all items, reasons for exclusion, factor loading and factor composition for the included items (*n* = 445).

N°	Item	Mean (SD)	Factor	F1	F2
1	Back pain sufferers should refrain from all physical activity in order to avoid injury.	1.9 (1.2)	E (B)		
2	Good posture prevents back pain.	4.2 (1.2)	E (B)		
3	Knowledge of the tissue damage is not necessary for effective therapy.	2.5 (1.4)	E (B)		
4	Reduction of daily physical exertion is a significant factor in treating back pain.	2.9 (1.4)	E (B)		
5	Not enough effort is made to find the underlying organic causes of back pain.	4.2 (1.3)	E (B)		
6	Mental stress can cause back pain even in the absence of tissue damage.	4.8 (1.4)	2		0.771
7	The cause of back pain is unknown.	3.4 (1.4)	E (B)		
8	Unilateral physical stress is not a cause of back pain.	3.2 (0.9)	E (B)		
9	Patients who have suffered chronic low back pain should avoid activities that stress the back.	3.7 (1.5)	1	0.614	
10	Pain is a nociceptive stimulus, indicating tissue damage.	3.3 (1.6)	1	0.621	
11	A patient suffering from severe low back pain will benefit from physical exercise.	4.8 (1.1)	2		0.701
12	Functional limitations associated with back pain are the result of psychosocial factors.	3.8 (1.1)	E (C)		
13	The best advice for low back pain is: ‘Take car’ and ‘Make no unnecessary movements’.	2.6 (1.5)	1	0.772	
14	Patients with back pain should preferably practice only pain-free movements.	3.5 (1.3)	E (B)		
15	Back pain indicates that there is something dangerously wrong with the back.	3.0 (1.4)	E (C)		
16	The way patients view their pain influences the progress of the symptoms.	4.7 (1.3)	2		0.529
17	Therapy may have been successful even if the pain remains.	3.5 (1.4)	E (B)		
18	Therapy can completely alleviate the functional symptoms caused by back pain.	4.1 (1.3)	E (B)		
19	If ADL activities cause more back pain, this is not dangerous.	2.4 (1.1)	E (B)		
20	Low back pain indicates the presence of organic injury.	2.8 (1.4)	1	0.738	
21	Sports should not be recommended for patients with back pain.	2.1 (1.2)	E (A)		
22	If back pain increases in severity, I immediately adjust the intensity of my treatment accordingly.	4.2 (1.4)	E (B)		
23	If therapy does not result in a reduction in back pain, there is a high risk of severe restrictions in the long term.	3.5 (1.3)	E (B)		
24	Pain reduction is a precondition for the restoration of normal functioning.	5.0 (1.2)	E (A)		
25	Increased pain indicates new tissue damage or the spread of existing damage.	2.9 (1.2)	1	0.677	
26	It is the task of the physiotherapist to remove the cause of back pain.	3.8 (1.4)	1	0.519	
27	There is no effective treatment to eliminate back pain.	3.0 (1.3)	E (B)		
28	TENS and/or back braces support functional recovery.	3.5 (1.3)	E (B)		
29	Even if the pain has worsened, the intensity of the next treatment can be increased.	2.7 (1.1)	E (B)		
30	If patients complain of pain during exercise, I worry that damage is being caused.	3.3 (1.4)	1	0.661	
31	The severity of tissue damage determines the level of pain.	3.3 (1.4)	1	0.731	
32	A rapid resumption of daily activities is an important goal of the treatment.	5.0 (0.8)	E (A)		
33	Learning to cope with stress promotes recovery from low back pain.	4.8 (1.0)	2		0.649
34	Exercises that may be back straining should not be avoided during the treatment.	4.4 (1.3)	2		0.547
35	In the long run, patients with back pain have a higher risk of developing spinal impairments.	3.1 (1.3)	E (B)		
36	In back pain, imaging tests are unnecessary.	3.4 (0.9)	E (B)		

SD: standard deviation; ADL: activities of daily living; TENS: transcutaneous electrical nerve stimulation; N°: number of items of the original questionnaire; E = exclusion item. Reason for exclusion A = non-heterogeneity (skewness); B = minimal loading; C = increases alpha if item deleted. Answer options: 1 = ‘*Totally disagree’*, 2 = ‘*Largely disagree’*, 3 = *‘Disagree to some extent’*, 4 = ‘*Agree to some extent’*, 5 = *‘Largely agree’*, 6 = ‘*Totally agree’*. Factors: F1 = biomedical factor; F2 = biopsychosocial factor.

**Table 3:** Sensitivity to change and effect size estimations of the PABS-PT-SP after pain educational training; *n* = 53.

		*t*-Test Paired	SRM [95% CI]			r	R^2^
Control group (*n* = 26)	BM factor	No statistical significance	—	Correlation between BM factor ---- BPS factor	Time 1 (BASELINE)	−0.47 *	0.22
	BPS factor	No statistical significance	—		Time 2 (POST-INTERVENTION)	−0.45 *	0.20
Experimental group (*n* = 27)	BM factor	*t* = 3.231; *df* = 26 *p*-value < 0.05 *	0.53 [0.22, 0.73]		Time 1 (BASELINE)	−0.44 *	0.19
	BPS factor	*t* = −3.567; *df* = 26 *p*-value < 0.001 **	0.76 [0.39, 0.98]		Time 2 (POST-INTERVENTION)	−0.68 **	0.46

BM factor: biomedical factor of the questionnaire; BPS factor: biopsychosocial factor of the questionnaire. SRM: standardized response mean; CI: confidence interval; *r*: Pearson’s correlation coefficient; *R^2^*: coefficient of determination. *: *p*-value < 0.05; **: *p*-value < 0.001.

**Table 4:** Comparison of studies assessing the factor structure and internal consistency of the PABS-PT.

	Factor 1 (Biomedical)			Factor 2 (Biopsychosocial)		
	Number of Items	Cronbach’s Alpha	Variance Explained (%)	Number of Items	Cronbach’s Alpha	Variance Explained (%)
Ostelo et al., 2003 [27]	14	0.84	25.2	6	0.54	8.2
Houben et al., 2005 [30]	10	0.73	23.4	9	0.68	10
Laekeman et al., 2008 [31]	10	0.77	21.5	4	0.58	3.6
Watson et al., 2008 * [34]	12	0,.79	-	5	0.60	-
Magalhaes et al., 2011 [15]	10	0.74	-	9	0.67	-
Dalkilinc et al., 2014 [32]	7	0.72	24.5	6	0.59	14
Mutsaers et al., 2014 ** [41]	7	0.75	-	8	0.73	-
Eland et al., 2016 [33]	13	0.79	18.1	6	0.57	7.1
Gacto-Sánchez et al., 2023 *** [72]	9	0.72	49.3	7	0.71	26.5
this work	8	0.86	39.4	5	0.77	13.8

*: General practitioners; **: physiotherapists’ attitudes and beliefs in neck pain; ***: physiotherapy students.

## Data Availability

Data are available from the corresponding author upon reasonable request.

## Supplementary Materials

The following supporting information can be downloaded at: https://www.mdpi.com/article/10.3390/jcm12186045/s1, Supplementary File S1: Summary of the selected items in each study on the PABS-PT., Supplementary File S2: Spanish version of PABS-PT., Supplementary File S3: Comparison of studies assessing psychometric properties of the PABS-PT in physiotherapists.
