# Supplementary material for: Transcultural Adaptation and Psychometric Validation of the Spanish Version of the Pain Attitudes and Beliefs Scale for Physiotherapists
Source: J Clin Med. 2023 Sep 19;12(18):6045. doi: 10.3390/jcm12186045 (PMC10531514; doi:10.3390/jcm12186045)
Supplement: Supplementary file 1 [file jcm-12-06045-s001.zip › Supplementary File S1.pdf]

	Ostelo *et al.*(2003)	Houben *et al.* (2005)	Laekeman *et al.* (2008)	Watson *et al.* (2008)*	Magalhaes *et al.* (2011)	Dalkilinc *et al.* (2015)	Mutsaers *et al.* (2014)**	Eland *et al.* (2016)	Gacto-Sánchez *et al.* (2023)***	Díaz-Fernández *et al.* (2023) Spanish Version
Language	Dutch	Dutch	German	English	Brazilian-Portuguese	Turkish	Dutch	Norwegian	Spanish	Spanish
Composition of the original scale	31 items	36 items	36 items	36 items	31 items	31 items	36 items	36 items	36 items	36 items
Number of items included for factor analysis	20	19	17	17	19	13	15	19	16	13
Factor composition of Factor 1 (Biomedical)	25, 24, 31, 26, 22, 30, 10, 13, 14, 9, 20, 5, 23, 4	31, 25, 10, 22, 30, 14, 24, 23, 20, 35	25, 31, 23, 10, 30, 26, 35, 4, 5, 14	31, 25, 10, 22, 30, 14, 24, 20, 35	31, 25, 10, 22, 30, 9, 24, 23, 20, 35	24, 23, 31, 25, 28, 14, 9	25, 23, 24, 29, 31, 10, 20	25, 20, 30, 31, 24, 10, 23, 14, 26, 4, 5, 35, 22	31, 25, 20, 10, 9, 2, 13, 15, 1	13, 20, 31, 25, 30, 10, 9, 26
Internal consistency Factor Biomedical	α = 0.83	α = 0.73	α = 0.77	α = 0.79	α = 0.74	α = 0.72	α = 0.75	α = 0.79	α = 0.72	α = 0.86
Factor composition of Factor 2	7, 12, 6, 3, 27, 11	33, 11, 29, 34, 17, 7, 12, 27, 6	34, 29, 19, 11	6, 7, 12, 27, 33	33, 11, 29, 34, 17, 7, 12, 27, 6	20, 15, 13, 12, 17, 30	12, 36, 17, 6, 7, 33, 27, 3	11, 33, 29, 34, 17, 22	33, 16, 6, 10, 32, 12, 29	6, 11, 33, 34, 16

(Biopsychosocial)										
Internal consistency Factor Biopsychosocial	α = 0.54	α = 0.68	α = 0.58	α = 0.60	α = 0.67	α = 0.59	α = 0.73	α = 0.55	α = 0.71	α = 0.77
Explained variance for Factor Biomedical (%)	25.2	23.4	21.5	-	-	24.5	-	18.1	49.3	39.4
Explained variance for Factor Biopsychosocial (%)	8.2	8.2	3.6	-	-	14	-	7.1	26.5	13.8

Table S1. Summary of items' selection from the studies on PABS-PT. **: General Practitioners; **: Physiotherapists' attitudes and beliefs in neck pain; ***: Physiotherapy students; -: No data available.* For clarity of presentation, items are sorted in descending order based on the magnitude of loadings on measured items in the factor.
