# Supplementary material for: Transcultural Adaptation and Psychometric Validation of the Spanish Version of the Pain Attitudes and Beliefs Scale for Physiotherapists
Source: J Clin Med. 2023 Sep 19;12(18):6045. doi: 10.3390/jcm12186045 (PMC10531514; doi:10.3390/jcm12186045)
Supplement: Supplementary file 1 [file jcm-12-06045-s001.zip › Supplementary File S2.pdf]

APPENDIX I: PABS-PT-Spanish Version

1. El estrés mental puede causar dolor de espalda incluso en ausencia de lesión tisular.
2. Los pacientes que han sufrido de dolor de espalda deben evitar actividades que la fuercen.
3. El dolor es un estímulo nociceptivo, indica daño en el tejido.
4. Los pacientes que sufren de intenso dolor lumbar pueden beneficiarse de la realización de ejercicios físicos.
5. Los mejores consejos para pacientes con dolor lumbar son: “Ten cuidado” y “No hagas movimientos innecesarios”.
6. La forma en que los pacientes piensan sobre su dolor influye en la evolución de los síntomas.
7. El dolor de espalda indica presencia de daño orgánico o tisular.
8. Un aumento del dolor indica nuevo daño tisular o extensión de la lesión ya existente.
9. Es tarea del fisioterapeuta eliminar la causa del dolor lumbar.
10. Si los pacientes se quejan de dolor lumbar durante el ejercicio o la actividad física, me preocupa que se estén lesionando.
11. La gravedad del daño en los tejidos determina el nivel de dolor.
12. Aprender a manejar el estrés promueve la recuperación del dolor de espalda.
13. Los ejercicios que puedan tensionar la espalda no deben ser evitados durante el tratamiento.

Opciones de respuesta:

1 = “Totalmente en desacuerdo”
2 = “Muy en desacuerdo”
3 = “Bastante en desacuerdo”
4 = “Bastante de acuerdo”
5 = “Muy de acuerdo”
6 = “Totalmente de acuerdo”

Factor Biomédico = 2 + 3 + 5 + 7 + 8 + 9 + 10 + 11
Factor Biopsicosocial = 1 + 4 + 6 + 12 + 13

La puntuación total de cada factor es la suma de todos los ítems que lo componen.

PABS-PT-Spanish Version

1. **El estrés mental puede causar dolor de espalda incluso en ausencia de lesión tisular.**

☐	☐	☐	☐	☐	☐
Totalmente en desacuerdo	Muy en desacuerdo	Bastante en desacuerdo	Bastante de acuerdo	Muy de acuerdo	Totalmente de acuerdo

2. **Los pacientes que han sufrido de dolor de espalda deben evitar actividades que la fuercen.**

☐	☐	☐	☐	☐	☐
Totalmente en desacuerdo	Muy en desacuerdo	Bastante en desacuerdo	Bastante de acuerdo	Muy de acuerdo	Totalmente de acuerdo

3. **El dolor es un estímulo nociceptivo, indica daño en el tejido.**

☐	☐	☐	☐	☐	☐
Totalmente en desacuerdo	Muy en desacuerdo	Bastante en desacuerdo	Bastante de acuerdo	Muy de acuerdo	Totalmente de acuerdo

4. **Los pacientes que sufren de intenso dolor lumbar pueden beneficiarse de la realización de ejercicios físicos.**

☐	☐	☐	☐	☐	☐
Totalmente en desacuerdo	Muy en desacuerdo	Bastante en desacuerdo	Bastante de acuerdo	Muy de acuerdo	Totalmente de acuerdo

5. **Los mejores consejos para pacientes con dolor lumbar son: "*Ten cuidado*" y "*No hagas movimientos innecesarios*".**

☐	☐	☐	☐	☐	☐
Totalmente en desacuerdo	Muy en desacuerdo	Bastante en desacuerdo	Bastante de acuerdo	Muy de acuerdo	Totalmente de acuerdo

6. **La forma en que los pacientes piensan sobre su dolor influye en la evolución de los síntomas.**

☐	☐	☐	☐	☐	☐
Totalmente en desacuerdo	Muy en desacuerdo	Bastante en desacuerdo	Bastante de acuerdo	Muy de acuerdo	Totalmente de acuerdo

7. **El dolor de espalda indica presencia de daño orgánico o tisular**.

☐	☐	☐	☐	☐	☐
Totalmente en desacuerdo	Muy en desacuerdo	Bastante en desacuerdo	Bastante de acuerdo	Muy de acuerdo	Totalmente de acuerdo

8. **Un aumento del dolor indica nuevo daño tisular o extensión de la lesión ya existente.**

☐	☐	☐	☐	☐	☐
Totalmente en desacuerdo	Muy en desacuerdo	Bastante en desacuerdo	Bastante de acuerdo	Muy de acuerdo	Totalmente de acuerdo

9. Es tarea del fisioterapeuta eliminar la causa del dolor lumbar.

☐	☐	☐	☐	☐	☐
Totalmente en desacuerdo	Muy en desacuerdo	Bastante en desacuerdo	Bastante de acuerdo	Muy de acuerdo	Totalmente de acuerdo

10. Si los pacientes se quejan de dolor lumbar durante el ejercicio o la actividad física, me preocupa que se estén lesionando.

☐	☐	☐	☐	☐	☐
Totalmente en desacuerdo	Muy en desacuerdo	Bastante en desacuerdo	Bastante de acuerdo	Muy de acuerdo	Totalmente de acuerdo

11. La gravedad del daño en los tejidos determina el nivel de dolor.

☐	☐	☐	☐	☐	☐
Totalmente en desacuerdo	Muy en desacuerdo	Bastante en desacuerdo	Bastante de acuerdo	Muy de acuerdo	Totalmente de acuerdo

12. Aprender a manejar el estrés promueve la recuperación del dolor de espalda.

☐	☐	☐	☐	☐	☐
Totalmente en desacuerdo	Muy en desacuerdo	Bastante en desacuerdo	Bastante de acuerdo	Muy de acuerdo	Totalmente de acuerdo

13. Los ejercicios que puedan tensionar la espalda no deben ser evitados durante el tratamiento.

☐	☐	☐	☐	☐	☐
Totalmente en desacuerdo	Muy en desacuerdo	Bastante en desacuerdo	Bastante de acuerdo	Muy de acuerdo	Totalmente de acuerdo

Factor Biomédico = 2 + 3 + 5 + 7 + 8 + 9 + 10 + 11
Factor Biopsicosocial = 1 + 4 + 6 + 12 + 13

La puntuación total de cada factor es la suma de todos los ítems que lo componen.
