# Supplementary material for: Transcultural Adaptation and Psychometric Validation of the Spanish Version of the Pain Attitudes and Beliefs Scale for Physiotherapists
Source: J Clin Med. 2023 Sep 19;12(18):6045. doi: 10.3390/jcm12186045 (PMC10531514; doi:10.3390/jcm12186045)
Supplement: Supplementary file 1 [file jcm-12-06045-s001.zip › Supplementary File S3.pdf]

	FACTOR STRUCTURE / INTERNAL CONSISTENCY						*RELIABILITY (ICC; 95% [CI])*		*MEASUREMENT ERROR INTERPRETABILITY (SEM; MDC_{95})*		*CONCURRENT CONVERGENT VALIDITY (r Pearson)*			*SENSITIVITY TO CHANGE RESPONSIVENESS t-test paired SRM (95% [CI])*		*PREDICTIVE ABILITY AUC (95% [CI]) Cut-off points*		*DISCRIMINATIVE ABILITY (Known-groups)*	
	BM factor			BPS factor			BM factor	BPS factor	BM factor	BPS factor	BM factor	BPS factor	Total score PABS-PT	BM factor	BPS factor	BM factor	BPS factor	BM factor	BPS factor
	Items number	Cronbach's alpha	Variance explained (%)	Items number	Cronbach's alpha	Variance explained (%)													
Ostelo *et al.*(2003)<br>PHYSIOTHERAPISTS	14	0.84	25.2	6	0.54	8.2	-	-	-	-	-	-	-	-	-	-	-	-	
Houben *et al.* (2005a, 2005b)<br>PHYSIOTHERAPISTS	10	0.73	23.4	9	0.68	10	-	-	-	-	0.51**	HC-PAIRS<br>-0.47**	-	-	-	-	-	-	
Laekeman *et al.* (2008)<br>PHYSIOTHERAPISTS	10	0.77	21.5	4	0.58	3.6	0.83<br>-	0.70<br>-	-	-	0.72**	TSK (Adapted German Version)<br>-0.54**	-	-	-	-	-	-	
Watson *et al.* (2008)<br>GENERAL PRACTITIONERS	12	0,.79	-	5	0.60	-	-	-	-	-	-	-	-	-	-	-	-	-	
Magalhaes *et al.* (2011)<br>PHYSIOTHERAPISTS	10	0.74	-	9	0.67	-	0.80<br>[0.72-0,87]	0.70<br>[0.57-0,94]	3.5<br>7.1 %	3.48<br>7.7 %	0.28**	HC-PAIRS<br>0.19**	0.55**	-	-	-	-	-	
Dalkilinc *et al.* (2014)<br>PHYSIOTHERAPISTS	7	0.72	24.5	6	0.59	14	0.81<br>[0.60-0,91]	0.82<br>[0.61-0,91]	-	-	0.29	TSK (Turkish Version)<br>- 0.29	0.39	-	-	-	-	-	
Mutsaers *et al.* (2014)<br>PHYSIOTHERAPISTS NECK PAIN	7	0.75	-	8	0.73	-	0.73<br>[0.56-0,83]	0.82<br>[0.71-0,89]	3.01<br>8.34	1.58<br>4.37	-	HC-PAIRS<br>-	0.55	-	-	-	-	-	
Eland *et al.* (2016)<br>PHYSIOTHERAPISTS	13	0.79	18.1	6	0.57	7.1	-	-	-	-	-	-	-	-	-	-	-	-	
Gacto-Sánchez *et al.* (2023)<br>PHYSIOTHERAPY' STUDENTS	9	0.72	49.3	7	0.71	26.5	0.85	0.7	2.17<br>6.01	2.01<br>5.56	-	-	-	-	-	-	-	*p < 001*<br>Hypothesis was confirmed with *'Previous pain training'* in BPS<br>*d* = 1.24	
Spanish versión	8	0.86	39.4	5	0.77	13.8	0.84<br>[0.72-0,91]	0.82<br>[0.67-0,89]				HC-PAIRS (Spanish Version)		*t* = 3.231<br>*gl* = 26<br>*p* <0.05*	*t* = – 0.3567<br>*gl* = 26<br>*p* <0.001**	0.74<br>(0.7-0.79)<br>19.5	0.69<br>(0.64-0.74)<br>23.5	Hyphothesis was confirmed only on the BM factor	

Díaz-Fernández *et al.* (2023) PHYSIOTHERAPISTS	3.9	2.4	0.48**	-0.57**	-	0.53 (0.22-0.73)	0.76 (0.39-0.98)	*'Previous pain training'* (*t* = - 2.266; *gl* = 443; *p* < 0.05*) *d* = 0.46 Not in the BPS factor
	8.8	5.5	R-NPQ (Spanish Version)					
			-0.38**	0.41**	-			

Table S2. Comparison of studies assessing psychometric properties of the PABS-PT in physiotherapists. *BM: Biomedical; BPS: Biopsychosocial; -: No data available; TSK: Tampa Scale of Kinesiophobia. HC-PAIRS: Health Care Providers' Pain and Impairment Relationship Scale; R-NPQ: Revised Neurophysiology Pain Questionnaire; *: p-value < 0.05; **: p-value < 0.01.*
